# Geometric and dimensional characteristics of simulated curved canals prepared with proTaper instruments

**DOI:** 10.1590/S1678-77572010000100009

**Published:** 2010

**Authors:** Renata de Castro MARTINS, Maria Guiomar de Azevedo BAHIA, Vicente Tadeu Lopes BUONO

**Affiliations:** 1 DDS, MSc, Postgraduate student, Department of Metallurgical and Materials Engineering, School of Engineering, Federal University of Minas Gerais, Belo Horizonte, MG, Brazil.; 2 DDS, MSc, PhD, Full Professor, Department of Restoration Dentistry, Dental School, Federal University of Minas Gerais, Belo Horizonte, MG, Brazil.; 3 BS, MSc, PhD, Full Professor, Department of Metallurgical and Materials Engineering, School of Engineering, Federal University of Minas Gerais, Belo Horizonte, MG, Brazil.

**Keywords:** Nickel-titanium, Endodontic instruments, Curved canals

## Abstract

**Objective:**

This study identified which regions of ProTaper instruments work during curved root canal instrumentation.

**Material and methods:**

Twelve ProTaper instruments of each type, S1, S2, F1, and F2, were assessed morphometrically by measuring tip angle, tip length, tip diameter, length of each pitch along the cutting blades, and instrument diameter at each millimeter from the tip. Curved canals in resin blocks were explored with manual stainless steel files and prepared with ProTaper instruments until the apical end following four distinct sequences of instrumentation: S1; S1 and S2; S1, S2, and F1; S1, S2, F1, and F2. Image analysis was employed for measuring canal diameters. The diameters of the canals and diameters of the instruments were compared. Data were analyzed by one-way ANOVA and Tukey’s test.

**Results:**

No statistically significant difference was found between the canals and instrument diameters (p>0.05). The largest diameters in the end-point of the instrumented canals were obtained with F1 and F2 instruments and in the initial and middle thirds with S1 and S2 instruments.

**Conclusions:**

All instruments worked at the tip and along their cutting blades, being susceptible to fail by torsion, fatigue, or the combination of these two mechanisms.

## INTRODUCTION

Failure of NiTi rotary endodontic instruments takes place under two circumstances: torsional overload and flexural fatigue fracture^14^. Torsional fracture takes place when the tip or any part of the endodontic instrument is locked in a canal while its shaft continues to rotate. In this case, the elastic limit of the metal is exceeded and it undergoes plastic deformation followed by fracture^4,14^. The fatigue life of a NiTi rotary instrument is related to its dimensions and to the degree in which it is flexed when placed in a curved root canal, with larger instrument diameters and greater flexures leading to a shorter expected life^2,10,12^.

According to their manufacturers, ProTaper nickel-titanium rotary instruments (Dentsply/ Maillefer, Ballaigues, Switzerland) were designed to improve cutting efficiency, flexibility, and safety, being developed for instrumentation of difficult, constricted, and severely curved canals with a few “shaping” and “finishing” instruments. The shaping instruments S1 and S2 have increasingly larger tapers over the length of their cutting blades, allowing each instrument to engage, cut, and prepare a specific area of the canal. One of the benefits of a progressively tapered shaping instrument is that each instrument engages a smaller zone of dentin, reducing torsional loads, file fatigue, and potential for breakage. The finishing instruments F1, F2, and F3 have fixed tapers between D_1_ and D_3_ and decreasing tapers from D_4_ to D_14_. This design feature serves to improve flexibility and safety by reducing the potential for taper-locking^13^.

The purpose of the present study was to evaluate the geometric and dimensional characteristics of simulated curved root canals in resin blocks prepared with ProTaper instruments, aiming to identify which regions of these instruments work during curved root canal instrumentation. This identification was complemented by scanning electron microscopy (SEM) observation of the instruments before and after canal instrumentation. Considering that the only information regarding the dimensions of ProTaper instruments is the one given by the manufacturer, and that morphometric variations have been reported among other NiTi rotary instruments^8,9^, the dimensions of ProTaper instruments were also evaluated, in an attempt to correlate these measurements with the geometric and dimensional characteristics of the curved root canals prepared with them.

## MATERIAL AND METHODS

**Figure 1:**
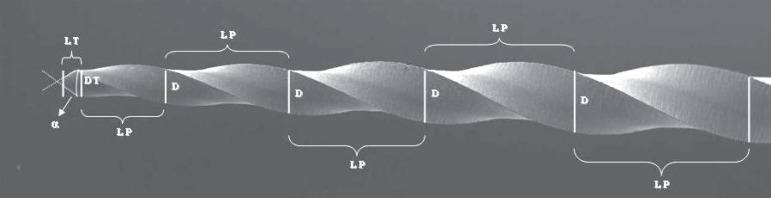
Morphometric parameters measured on the instruments according to ANSI/ADA Specification No. 101: tip angle (α), length of the tip (LT), diameter of the tip (DT); length of each pitch along the cutting blades (LP), and instrument diameter at each millimeter from the tip (D)

Twelve ProTaper instruments of each type, S1, S2, F1, and F2, were used, totalizing 48 instruments, which were examined with a microscope (Mitutoyo TM 500, Tokyo, Japan) at ×30 magnification to evaluate: angle of tip (α), length of tip (LT), diameter of tip (DT), length of each pitch along the cutting blades (LP), and instrument diameter (D) at each millimeter from the tip (Figure 1), based on the ANSI/ADA Specification Nº. 101^1^. By instrument diameter, it is meant the largest distance between its extremities in the section perpendicular to the long axis. Tip angle is the angle between two imaginary lines tangent to the tip edges.

Twenty-one curved root canals in transparent resin blocks (Dentsply/Maillefer), with 16 mm in length, radius of curvature of 3.5 mm and an angle of 53º, measured according to the method described by Pruett, et al.^12^ (1997), were employed. The point of maximum flexure was at 3.5 mm from the apical end. The canals were instrumented by the same operator, being firstly explored with sizes 10 and 15 stainless steel Kfiles up to their full length. One explored canal served as control. During instrumentation, the canals were irrigated with water and no lubricant was employed. The other 20 canals were prepared with ProTaper instruments up to their full length following 4 distinct instrumentation sequences (5 canals for each sequence): S1; S1 and S2; S1, S2, and F1; S1, S2, F1, and F2. The instruments were operated at 300 rpm, using a slow-speed high-torque endodontic electric motor (TC Motor 3000; Nouvag, Goldach, Switzerland), with a 16:1 gear reduction hand piece (W&H 975; Dentalwerk, Bürmoos, Austria). Canal shaping followed the manufacturer’s recommendations and the end of the canal was reached only once, the instruments staying there for no longer than 1 second^13^. After each instrumentation sequence the instruments were inspected with the microscope at ×30 magnification to evaluate any distortion on the cutting blades.

The canals were then photographed in a standardized manner, with a scale in millimeters, using a digital camera (Cyber-shot DSC - W1; Sony, Tokyo, Japan). The digital images were analyzed using Image-Pro Plus 6.0 imageanalysis software (Media Cybernetics, Silver Spring, MD, USA). The canals had their diameters measured from the full end of the canal upwards to the initial third, in 1 mm intervals. Before and after instrumentation of the canals, the instruments were examined in a scanning electron microscope (JSM 6360; JEOL, Tokyo, Japan). The diameters of the canals and the instruments were analyzed statistically by oneway ANOVA at a significance level of 5%.

## RESULTS

All ProTaper instruments examined presented a conical guide tip, with an average angle of 65.8 ± 2.25 degrees. The greatest variations in the tip angle were found in S1 (66.3 ± 2.91 degrees) and S2 (65.5 ± 2.68 degrees) instruments, while F1 (65.0 ± 1.49 degrees) and F2 (66.3 ± 1.52 degrees) instruments presented minor variations. Instruments S1 and S2 had 15 mm of active part, while F1 and F2 had 17 mm and 16 mm, respectively. The pitch length increased from the tip upwards in all instruments. The S1 and S2 instruments presented more spaced pitches along the cutting blades than the F1 and F2 instruments. S1, F2, and F3 instruments increased 45% on the first pitch length, and S2 and F1 instruments showed a smaller increase, around 35% and 23%, respectively. The increase in length from the second to the 8th pitches in S1 and S2 instruments was 8.5% and 12%, respectively. From the eighth to the last pitch, these instruments showed an increase of 21.5%. F1, F2, and F3 instruments presented an increase in length from the second to the last pitch of around 11, 12, and 10%, respectively.

**Figure 2:**
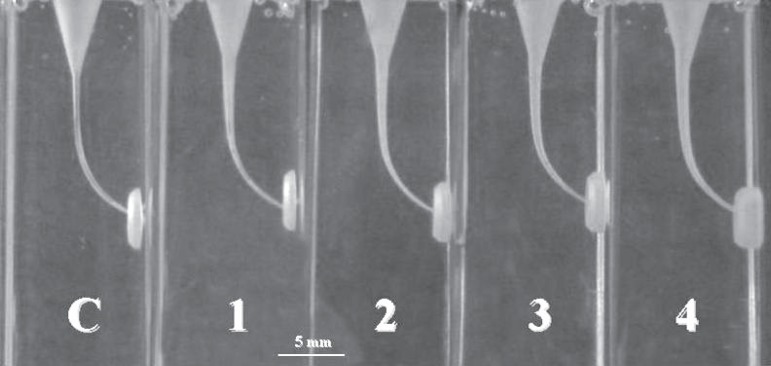
Final aspect of curved root canals in resin blocks instrumented until the end of the canal according to the 4 distinct sequences: Control (C); S1 (1); S1 and S2 (2); S1, S2, and F1 (3); and S1, S2, F1, and F2 (4)

The final aspect of 5 curved root canals in resin blocks instrumented according to the four distinct sequences used in this study is shown in Figure 2. These sequences developed a funnel shaped form^16^, with smallest diameter at the end-point and the widest diameter at the orifice.

The use of resin blocks was chosen instead of extracted teeth because they allow direct visualization of the preparation shape in the clear resin. In addition, the use of canals with defined shapes in a standardized way favors the assessment and precision of the measurements. However, because of the difference in hardness between dentin and the resin blocks^5^, care should be taken in extrapolating the results to the clinical situation.

**Figure 3:**
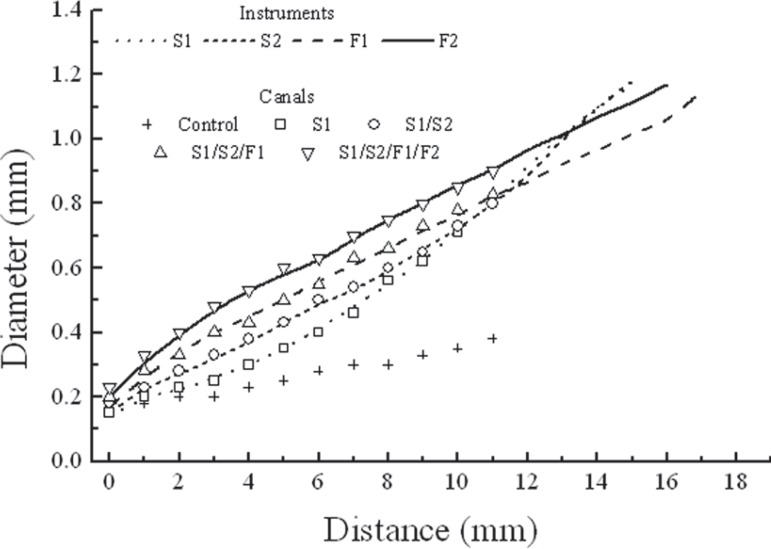
Mean diameters of the instrument measured at the tip and at each millimeter from the tip, and mean diameters of the canals measured from the end-point after hand instrumentation (control), and each of the different sequences of instrumentation employed

Figure 3 shows a plot of the mean diameters of the instruments measured at the tip and at each millimeter from the tip, as a function of tip distance, as well as the points representing the mean diameters of the canals measured from the end-point after each of the four sequences of instrumentation (standard deviations smaller than 5% of the mean diameter values). Figure 3 also shows that S1 instruments had a more gradual increase in diameter until D_10_. From D_11_ to D_15_, this increase in diameter was larger. In S2 instruments, the gradual increase in diameter goes until D_10_ and, from D_11_ to D_15_, the increase in diameter becomes larger. F1 and F2 instruments show the largest increase in diameter from D_1_ to D_3_. From D_4_ to the end of the active part, the increase in diameter in F1 and F2 becomes more gradual. These aspects can be observed in Figure 3 by the trajectory and inclination of the straight line corresponding to the ProTaper instruments. The close proximity of the lines (instrument diameters) and the data points (canal diameters) in Figure 3 indicates that the diameters obtained in the canals are closely related to the dimensions of the last instrument used in each sequence. One-way ANOVA showed no statistically significant difference among the diameters of the canals or the instruments (p>0.05). It can also be observed that all instruments worked in the end-point, middle portion and orifice of the canals. The largest diameters next the end-point were produced with F1 and F2 instruments, while most of the shaping of the orifice and middle portion of the canals was carried out by S1 and S2 instruments.

**Figure 4:**
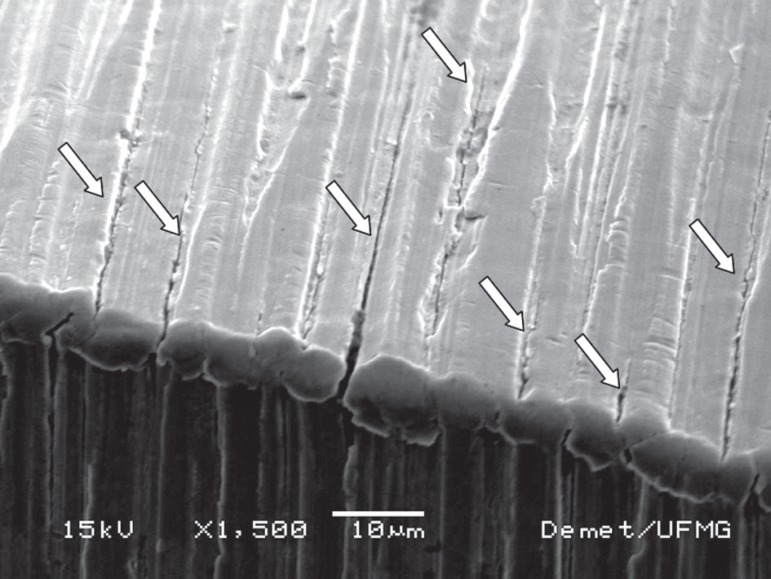
Cracks (arrows) on the cutting edge at 3.5 mm from the tip of an F1 instrument after canal shaping

**Figure 5:**
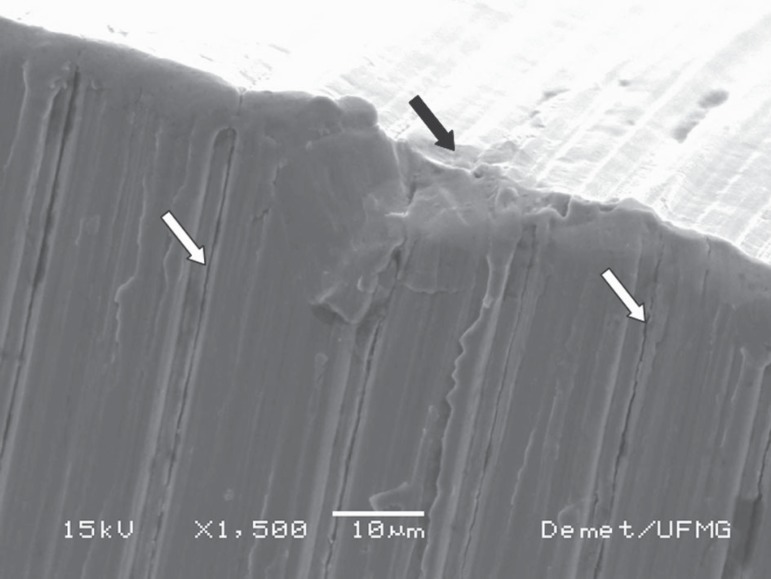
Cracks (white arrows), fretting mark, and smoothed surface (black arrow) at 7 mm from the tip of an S2 instrument after canal shaping

During canal shaping, no instrument separation occurred and no distortion was noticed when the instruments were observed by optical microscopy. However, all instruments inspected by SEM after canal shaping had microcracks, with a tendency of concentration of larger and wider cracks in the region D_3_ to D_4_, as illustrated in Figure 4 for an F1 instrument. In S1 and S2 instruments, finer microcracks, as well as signs of fretting, smoothed, or scratched surfaces were found until D_7_ (Figure 5).

## DISCUSSION

International standards are provided to establish manufacturer’s guidelines followed to produce consistent and reliable dental products. ANSI/ADA Specification Nº. 101^1^ does not strictly apply to ProTaper instruments because they show multiple tapers. However, it serves as a basis to measure geometric characteristics such as diameter at each millimeter from the tip, length of each pitch along the cutting blades, tip angle, tip length, and tip diameter. Since machining NiTi endodontic instruments is a complex procedure, morphometric variations among instruments of the same size have been reported^8,9^. In the present study, the largest variations in the tip angle were found in S1 and S2 instruments, probably because of the difficulty in machining instruments with small tip diameters. Despite of these variations, the tip angle of the instruments examined are in accordance with ANSI/ADA Specification Nº. 101^1^.

The S1 and S2 instruments presented more spaced pitches along the cutting blades and this may improve flexibility. Increasing the pitch also reduces the helical angle, decreasing the torsional load and the tendency to taper-locking^7^. The less spaced pitches in the initial part of F1 and F2 instruments promote greater resistance in this region, allowing these instruments to safely shape the apical third of the canal, while the steeper increase in pitch length restores instrument flexibility away from the tip. The variations in instrument diameter reported here complement this design: the larger increase in diameter in S1 from D_8_ to D_15_ makes this instrument adequate to shape the coronal third of the canal, while in S2 the increase in diameter is larger from D_5_ to D_10_, since this instrument was developed to enlarge the middle third of the canal; the F1 and F2 instruments, developed to shape the apical third of the canal, show the largest increase in diameter until D_3_. All instruments analyzed presented tip diameter and dimensions along the cutting blades smaller than those reported by the manufacturer and the literature^6,13^.

The mechanical properties of polymers differ from those of dentin is a well known fact, but this study investigates the geometry and dimension of canals after shaping, irrespectively of the efforts made during shaping. The approach used would not be feasible in extracted teeth because of lack of standardization on initial geometry and dimensions.

Comparing the canal diameters measured after each of the four instrumentation sequences employed with the control group diameters (Figure 3), it can be observed that all instruments worked close to their tips and along their cutting blades. Similar results were reported for ProFile instruments used in resin blocks^15^, but no such type of analysis could be found for ProTaper instruments. It can also be observed that the largest diameters next to the end-point were obtained with F1 and F2 instruments working until the full length, while most of the shaping of the orifice and middle portions was carried out by S1 and S2 instruments. However, S1 and S2 instruments also enlarge progressively the end third of the canals. In fact, Peng, et al.^11^ (2005) evaluated S1 instruments discarded after clinical use and found that these instruments fractured at a mean distance of 3.67 mm from their tips.

In the present study, SEM images revealed that the simulated clinical use of the ProTaper instruments produced alterations on their surfaces. Instruments S1 and S2 showed cracks next to the region of maximum canal curvature and in regions more distant from the tip. These results suggest that the instruments S1 and S2 are subjected to torsional stresses in the coronal and middle thirds because of their largest diameter in these regions, besides flexural stresses at the tip and at the curvature of the canals in the apical region, being thus prone to fail by two distinct mechanisms: fatigue in the apical portion and overloading in torsion in the coronal and middle thirds. It must be remembered that S1 and S2 instruments are the first to work in the whole extension of the canal, and thus their tip should act in curved canals whose apical portion is not adequately widened. The F1 and F2 instruments were developed to shape the apical third, but they also expand the shape into the middle and coronal thirds of the canal^6,13^. These instruments present a decrease in their lifetime in relation to S1 and S2 instruments because they work actively in the apical third of the canals, being thus subjected to higher deformation amplitudes due to the curvature of the canals associated with their largest diameter next to the tip^3^. In the present work, SEM analysis revealed a greater incidence of microcracks on the surface of these instruments close to the region of maximum canal curvature, that is, between 3 and 4 mm from their tip. Thus, it can be inferred that F1 and F2 instruments are subjected to torsional and flexural stresses in the apical third of the canals because of their largest diameter in this region and the curvature of the canals, being prone to fail by torsion, fatigue or by the combination of these two mechanisms.

## CONCLUSIONS

The largest diameters in the end-point of the instrumented canals were obtained with F1 and F2 instruments, while most of the shaping in the initial and middle thirds was performed by S1 and S2 instruments. However, all instruments worked at the tip and along their cutting blades, being prone to fail by torsion, fatigue, or by the combination of these two mechanisms.
